# Learning Mass Casualty Triage via Role-Play Simulation

**DOI:** 10.7759/cureus.92811

**Published:** 2025-09-20

**Authors:** Nicole Vuong, Martin Morales-Cruz, Drake Dixon, Ayanna Walker, Latha Ganti, Shayne Gue

**Affiliations:** 1 Emergency Medicine, Hospital Corporation of America (HCA) Florida Osceola Hospital, Kissimmee, USA; 2 Medical Science, The Warren Alpert Medical School of Brown University, Providence, USA; 3 Research, Orlando College of Osteopathic Medicine, Winter Garden, USA; 4 Emergency Medicine and Neurology, University of Central Florida, Orlando, USA; 5 Medical Education, University of Central Florida College of Medicine, Orlando, USA; 6 Emergency Medicine, BayCare Health System, St. Joseph's Hospital, Tampa, USA

**Keywords:** disaster medicine, event medicine, gamification, interactive learning, mass casualty incident, medical simulation, start

## Abstract

Background

The purpose of this educational intervention was to introduce trainees to the core competencies of disaster preparedness/resource allocation/mass casualty incident (MCI) command and event medicine. This innovative learning activity involving trainees from different programs teaches effective techniques of how to apply triage algorithms, such as Simple Triage and Rapid Treatment (START), in a mass casualty event.

Educational objectives

After participation in this educational session, learners were expected to be able to define a mass casualty incident (MCI) and discuss the unique challenges inherent to mass casualty incidents and disaster/event medicine, differentiate between day-to-day triage and triage during a mass casualty incident, and apply the components of Simple Triage and Rapid Transport (START) for mass casualty incidents.

Curricular design

The scenario was a music festival. A group of residents were granted backstage access to tour the concert grounds and medical tent. During the facility tour, the operations director (Proctor #2) radios the tour guide (Proctor #1) to let them know of an emergency crowd stampede due to unapproved pyrotechnics causing a fire; the medical tent suddenly became flooded with patients. “Patients” were trainees who received a laminated card labeled with vital signs and mental status and were transported one at a time to the tent. Residents ran over to the tent, performed triage, and then selected two of the most critical patients for air transport. The station leader documented the accuracy of each team. Winners were selected based on time of completion and accuracy of correctly triaging patients. For every incorrect triage, a 30-second penalty was added. Incorrectly triaged patient cards were debriefed in detail.

Impact/effectiveness

This activity engages learners both physically and mentally, necessitating everyone to be active. The impact was measured by a post-activity survey, accessed via QR at the station. Of the participants, 93% reported feeling better prepared to manage a real-life MCI. Further, 98% reported that START triage better motivated them to learn. In addition, 96% reported that this activity challenged them more than other learning activities. Verbal feedback included appreciation for the innovative activity design and being able to get some exercise.

## Introduction

Mass casualty incidents (MCIs) are defined by the World Health Organization as “disasters and major incidents characterized by quantity, severity, and diversity of patients that can rapidly overwhelm the ability of local medical resources to deliver comprehensive and definitive medical care” [1]. MCIs are not defined by the sheer volume of patients, but rather by the extent to which the event exceeds the resources and capabilities available within the local community [2]. These events can include natural disasters, terrorist violence, and transportation accidents. In the wake of events, such as the Columbine school shooting in 1999, the terrorist attacks on September 11, 2001, and Hurricane Katrina in 2005, the federal government has emphasized the need for preparedness by healthcare facilities and regional trauma systems to handle these resource-overwhelming incidents [2]. A key component of national preparedness is exercises or “drills,” to be performed regularly, as outlined by the US Department of Homeland Security Exercise and Evaluation Program (HSEEP) [3]. HSEEP provides guidelines to aid in the development and execution of exercises to evaluate the preparedness of the community for mass casualty events.

Emergency medicine physicians are the first point of medical contact in hospital settings and are at the forefront of healthcare systems. Increasing numbers of board-certified emergency medicine physicians are taking on leadership roles in prehospital and emergency medical services (EMS), thereby directly participating in (and leading) disaster planning and management [4]. As of 2023, the National Association of EMS Physicians reports there are 77 Accreditation Council for Graduate Medical Education (ACGME)-accredited EMS fellowship programs in the United States [5]. However, not all emergency medicine physicians choose to subspecialize in EMS; thus, emergency medicine residencies should include dedicated training in disaster medicine as they remain fundamental in disaster response teams in the event of an MCI.

Traditionally, lectures, simulations, and hospital-wide or regional drills have been utilized as the main educational methods. Gamification is a different educational strategy that uses elements of game design in non-game contexts and has increased in popularity and usage in recent years [6]. As disaster medicine continues to evolve, educational strategies that integrate active, scenario-based learning have shown effectiveness in building competence and confidence during emergency response [7]. Simulation-based training has been shown to improve triage accuracy during mass casualty incidents, particularly when used to mimic high-pressure scenarios such as pediatric disaster drills [8]. Similar initiatives, such as training with the Simple Triage and Rapid Treatment (START) system, which remains one of the most widely used triage algorithms, have demonstrated improved learner awareness, understanding, and successful application in mass casualty preparedness education [9]. In this project, the designers sought to evaluate whether a gamified training session would enhance the educational experience for residents and students applying disaster medicine concepts.

This article was previously presented as a meeting abstract at the 2023 Council of Residency Directors in Emergency Medicine (CORD) Academic Assembly on March 22, 2023.

## Materials and methods

This gamified triage application station was part of a larger collaborative event between two community-based emergency medicine residency programs in Orlando, Florida. “Disaster Day” was a music festival-themed disaster training event hosted by the University of Central Florida College of Medicine, providing disaster medicine education for 49 residents and students from both programs [4].

At this station, the educational objectives included the following: define a mass casualty incident (MCI) and discuss the unique challenges inherent to mass casualty incidents and disaster/event medicine, differentiate between day-to-day triage and triage during a mass casualty incident, and apply the components of Simple Triage and Rapid Transport (START) for mass casualty incidents. The three educational objectives were assessed using a combination of observed performance during the station and a structured post-session survey. Objective 1 (define an MCI and discuss unique challenges) and objective 2 (differentiate between day-to-day triage and MCI triage) were primarily assessed through learner self-report on the post-session survey, which included an item on perceived preparedness to manage a real-life MCI. Objective 3 (apply the components of the START algorithm) was assessed through direct observation and scoring of team performance. Each team’s accuracy in assigning triage tags and prioritizing patient transport was recorded by faculty proctors, and penalties were applied for incorrect triage decisions. Thus, while objectives 1 and 2 were measured by learner perceptions of preparedness and confidence, objective 3 was measured through objective task completion (accuracy and timing). The full survey instrument, including exact question wording and Likert scale response options, is included in the Appendices.

In this triage scenario, participants took on the role of volunteers working in the medical tent of a large music festival, granting them backstage access and a tour of the festival grounds. During their facility tour, the operations director suddenly radioed the tour guide to notify them of a fire caused by unauthorized pyrotechnics, leading to a massive crowd stampede. Learners then had to quickly coordinate among themselves to assign roles. Roles included locating victims and assigning triage tags, transporting victims to the medical tent, and prioritizing the transport of the most severely injured patients to local facilities.

**Figure 1:**
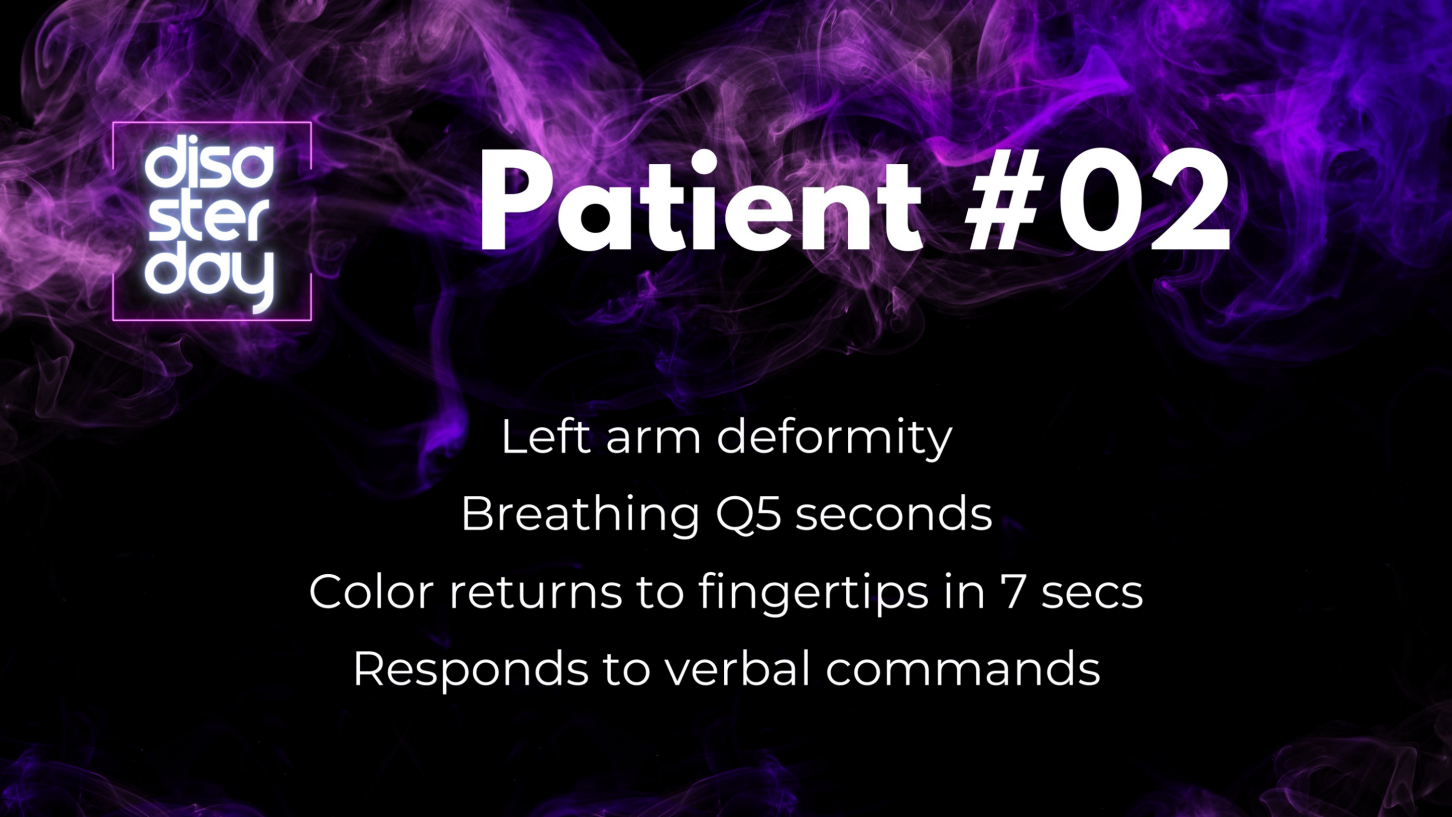
Example of a patient triage card

Team members assigning triage tags were tasked with running into the field to find all the “patients” (20 in total), which were represented by laminated sheets of paper that provided a brief patient synopsis with vital signs and mental status (Figure 1). Learners then determined which colored tag was appropriate to assign (green, yellow, red, or black), following the common triage algorithms.

Team members transporting victims were tasked with transporting patients one by one to the medical tent, determining which patients to transport first based on their triage tag and provided information.

Team members at the medical tent evaluated all patients brought to the tent and determined which patients required airlifting versus ground transport to a facility, based on limited resource availability. Further, they determined the order in which patients needed to be transported.

The proctors for this station were also some of the authors of this manuscript. Prior to the event, they reviewed the START triage algorithm, station flow, and scoring procedures to ensure consistency. The scenario was standardized across all teams, utilizing the same 20 laminated patient cards developed from established START templates and distributed consistently within the field area. The physical layout included a designated field for locating patients, a pathway for transport, and a central medical tent. Teams were scored based on timing and accuracy. The pre-brief for the station was untimed. Time officially started when members of the team were sent into the field and ended once patients requiring transport by air were identified and transport was called for. A 30-second penalty was applied for every incorrect triage, and the winner was determined based on the overall fastest time. All incorrect triages were debriefed in detail at the conclusion of the station after time had stopped. Scoring was performed by the same proctor for all groups, eliminating variability in scoring across teams.

This station utilized a cross-sectional survey design that evaluated the perceptions of emergency medicine residents and medical students on the effectiveness of the gamified strategy for learning disaster triage algorithms, compared to traditional learning methods. Specifically, participants were asked to complete a four-question post-session survey evaluating learner perceptions of motivation, engagement, challenge, and overall confidence in their ability to manage a real-life MCI compared to more traditional learning methods. The full survey instrument is included in the Appendices.

## Results

**Figure 2:**
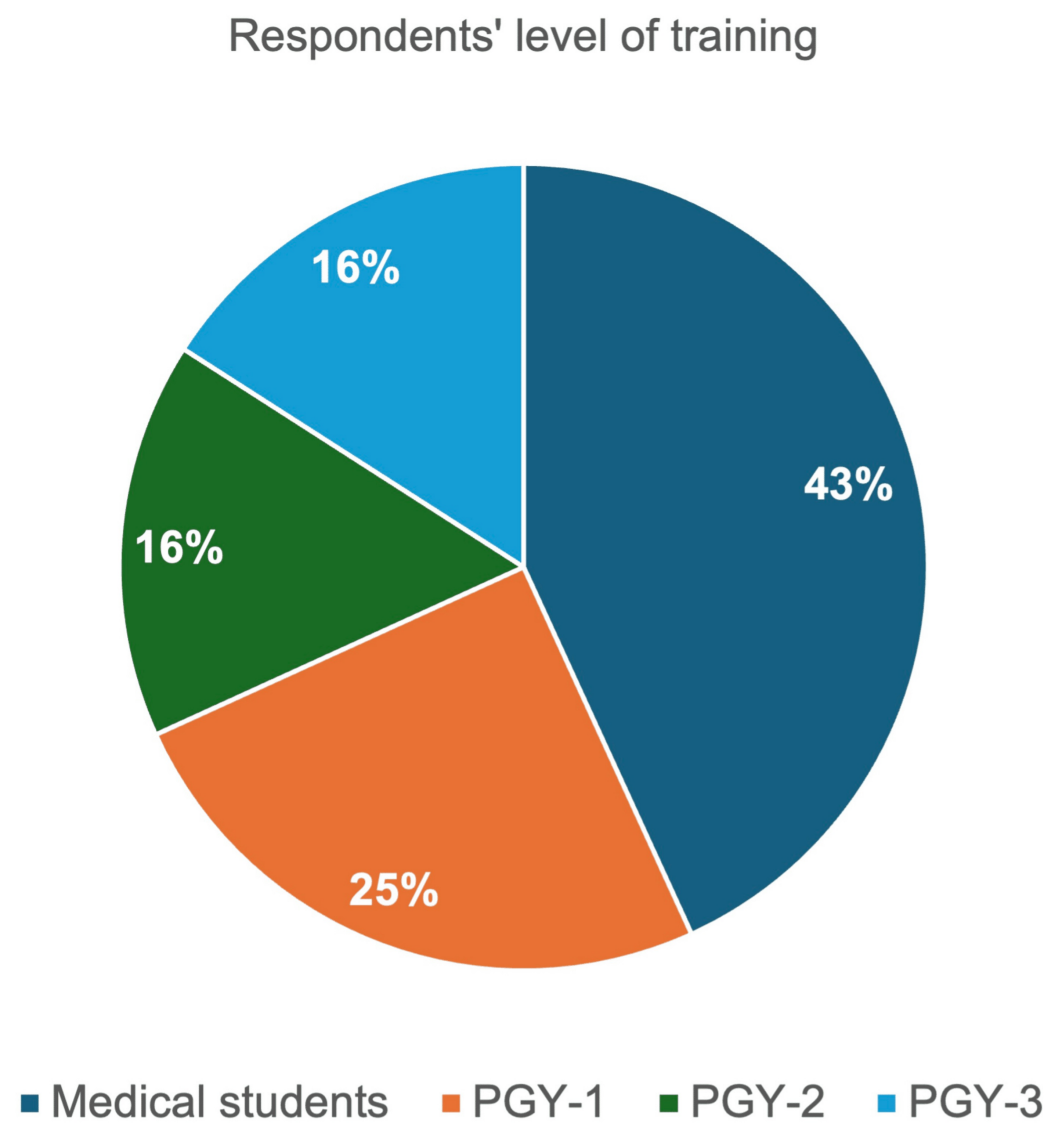
Respondents’ level of training PGY: postgraduate year of residency training

Of 49 total participants, 44 learners completed the post-session survey, including 19 medical students, 11 postgraduate year (PGY)-1s, seven PGY-2s, and seven PGY-3s (Figure 2).

**Figure 3:**
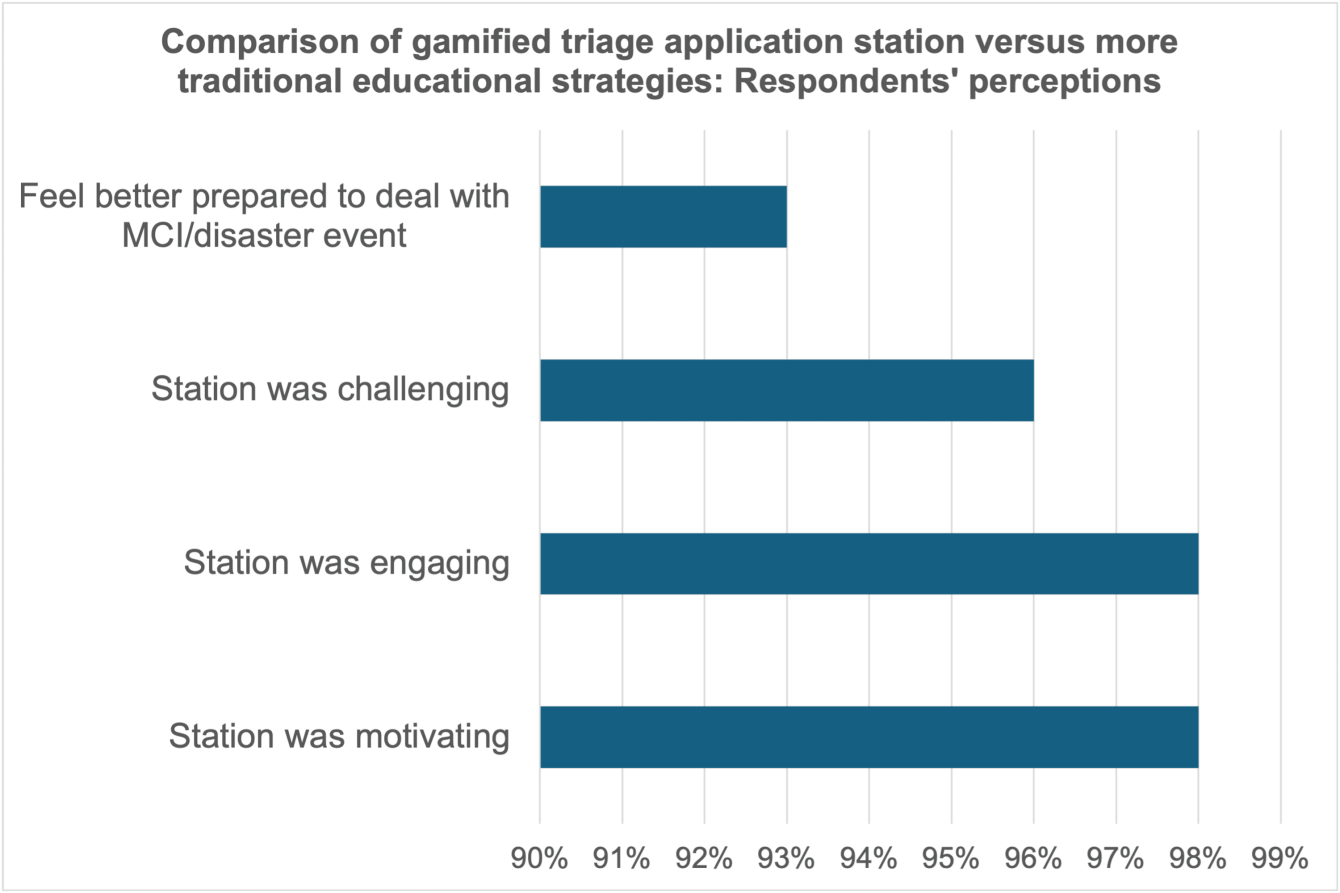
Respondents’ perception of the gamified triage application station compared to more traditional educational strategies MCI: mass casualty incident

Survey responses demonstrated that learners from all training levels perceived the gamified strategy to be effective. Respondents overwhelmingly “agreed” or “strongly agreed” that the station was more motivating (43 of 44, 98%), engaging (43 of 44, 98%), and challenging (42 of 44, 96%) than more traditional educational strategies. Further, 41 of 44 learners (93%) reported that the gamified station better prepared them to manage a real-life MCI/disaster/event medicine scenario as a result of their participation (Figure 3).

## Discussion

Emergency medicine physicians are generally the first point of medical contact for patients in hospital settings. Increasing numbers of EM physicians are taking on leadership roles in the prehospital setting and within EMS systems. Competency in the domain of event medicine and disaster management is a critical skill for all emergency medicine physicians. Specifically, the Model of the Clinical Practice of Emergency Medicine indicates that all emergency medicine physicians should be skilled in disaster management, including “preparedness, triage, mitigation, response, and recovery” [1]. Graduate medical education programs have traditionally relied on lectures, simulations, and/or hospital-wide drills. With the increased utilization of educational gamification, we sought to evaluate this teaching method in our disaster medicine curriculum.

We developed a novel music festival-themed “Disaster Day” competition event that included this triage gamification station, challenging learners to utilize the common triage algorithms to accurately assign triage tags and prioritize transport to appropriate facilities via appropriate transportation methods. This activity allowed participants to apply their medical knowledge and skills in a collaborative setting, with friendly competition among teams. Learners overwhelmingly indicated that the gamification element was motivating, engaging, and challenging compared to traditional learning methods. This indicates that educational gamification strategies may have broader applications and implications among learners of all levels.

This study has several strengths. The activity incorporated a novel, music festival-themed design that created a realistic and high-pressure environment for applying START triage. Learners engaged in both physical and cognitive tasks, which fostered teamwork and strong participation. The station was standardized across all groups, with identical patient scenarios and scoring conducted by the same proctor, which eliminated variability in scoring across teams. The survey response rate was high, and participants represented two separate residency programs, enhancing the generalizability of the findings beyond a single center.

Limitations should also be noted. Outcomes were restricted to learner perceptions (Kirkpatrick Level 1), as time and logistical constraints during the larger “Disaster Day” event did not allow for additional pre-/post-knowledge testing or evaluation of behavior change at individual stations [4,10]. While the use of a single proctor eliminated inter-rater variability, it also introduced the possibility of systematic bias if any errors in scoring occurred. In addition, the dual role of the authors as both proctors and investigators may have contributed to social desirability bias, although survey anonymity was maintained. Finally, the overall sample size remained modest despite the inclusion of two programs. Future work should expand to larger, multi-institutional cohorts and incorporate higher-level outcomes to better understand the impact of gamification on knowledge retention, skill acquisition, and performance.

## Conclusions

The triage gamification station was one component of a larger, novel gamification/competition event designed to educate emergency medicine residents and medical students on the core topics of mass casualty and disaster management. Our findings support gamification as a motivational technique incorporated into disaster training, evidenced by survey results indicating agreement with statements regarding enhanced motivation, engagement, and challenge compared to more traditional learning methods. Learners also reported improved confidence in their ability to manage real-life mass casualty incidents. While our study was limited to level one of Kirkpatrick’s model and limited by survey methodology and a small participant pool, we believe this adds to the growing body of literature supporting the continued study and utilization of gamified educational strategies across all educational domains.

## Appendices

Post-session survey

Table 1 presents the patient demographic questionnaire.

**Table 1:** Participant demographics PGY: postgraduate year of residency training

Which describes you?	
☐ Medical student
☐ PGY-1
☐ PGY-2
☐ PGY-3
☐ Attending physician
☐ Other: ____________________

Table 2 shows the survey instrument used in the present study. 

**Table 2:** Survey statements MCI: mass casualty incident

Statement	5 (strongly agree)	4 (agree)	3 (neutral)	2(disagree)	1 (strongly disagree)
Defining motivation as your desire or willingness to participate in an activity, triage gamification better motivated me to learn compared to other educational methods.	☐	☐	☐	☐	☐
Defining engagement as the level of meaningful involvement in an activity, triage gamification was more engaging compared to other educational methods.	☐	☐	☐	☐	☐
Defining challenge as the capacity of an activity to test your abilities, triage gamification better challenged me compared to other educational methods.	☐	☐	☐	☐	☐
I feel better prepared to manage a real-life MCI/disaster/event medicine scenario as a result of my participation in this activity.	☐	☐	☐	☐	☐
